# Synaptic depression and slow oscillatory activity in a biophysical network model of the cerebral cortex

**DOI:** 10.3389/fncom.2012.00064

**Published:** 2012-08-28

**Authors:** Jose M. Benita, Antoni Guillamon, Gustavo Deco, Maria V. Sanchez-Vives

**Affiliations:** ^1^Department of Applied Mathematics I - EPSEB, Universitat Politècnica de CatalunyaBarcelona, Spain; ^2^Department of Technology, Computational Neuroscience ICREA, Universitat Pompeu FabraBarcelona, Spain; ^3^ICREA-IDIBAPS (Institut d'Investigacions Biomèdiques August Pi i Sunyer)Barcelona, Spain

**Keywords:** cortical activity, network model, short-term depression, up/down state, synaptic plasticity

## Abstract

Short-term synaptic depression (STD) is a form of synaptic plasticity that has a large impact on network computations. Experimental results suggest that STD is modulated by cortical activity, decreasing with activity in the network and increasing during silent states. Here, we explored different activity-modulation protocols in a biophysical network model for which the model displayed less STD when the network was active than when it was silent, in agreement with experimental results. Furthermore, we studied how trains of synaptic potentials had lesser decay during periods of activity (UP states) than during silent periods (DOWN states), providing new experimental predictions. We next tackled the inverse question of what is the impact of modifying STD parameters on the emergent activity of the network, a question difficult to answer experimentally. We found that synaptic depression of cortical connections had a critical role to determine the regime of rhythmic cortical activity. While low STD resulted in an emergent rhythmic activity with short UP states and long DOWN states, increasing STD resulted in longer and more frequent UP states interleaved with short silent periods. A still higher synaptic depression set the network into a non-oscillatory firing regime where DOWN states no longer occurred. The speed of propagation of UP states along the network was not found to be modulated by STD during the oscillatory regime; it remained relatively stable over a range of values of STD. Overall, we found that the mutual interactions between synaptic depression and ongoing network activity are critical to determine the mechanisms that modulate cortical emergent patterns.

## Introduction

The cortical network is permanently active. Recurrent connections within cortical microcircuits, as well as connectivity with thalamus, hippocampus, interareal, and with other nuclei assure a constant bombardment of synaptic activity in any cortical state. Local cortical synaptic transmission exhibits different forms of plasticity, one of them being short-term synaptic depression (STD). STD can be defined as the decrease in the postsynaptic potentials following repetitive stimulation of a synapse. This process is directly related to the probability of release [for a review see Zucker and Regehr (2002)], and is a property of numerous intracortical synapses both excitatory and inhibitory (Thomson, 1997; Galaretta and Hestrin, 1998; Varela et al., 1999). STD has been reported in the cerebral cortex *in vitro* (Gil et al., 1997; Tsodyks and Markram, 1997; Varela et al., 1997; Thomson and Bannister, 1999; Reig et al., 2006). Both thalamocortical and intracortical synaptic connections from different areas of the cortex also exhibit STD *in vivo* (Chung et al., 2002; Petersen et al., 2003; Boudreau and Ferster, 2005; Reig et al., 2006; Reig and Sanchez-Vives, 2007; Stoelzel et al., 2008), although differences between *in vivo* and *in vitro* have also been reported (Borst, 2010).

Experimental and theoretical studies have suggested that STD plays various critical roles in cortical information processing. Some of these roles refer to sensory processing including visual cross-orientation suppression (Lauritzen et al., 2001; Carandini et al., 2002; Freeman et al., 2002), temporal characteristics of visual responses (Chance et al., 1998), sound localization (Cook et al., 2003), and adaptation to repetitive visual (Nelson, 1991a,b,c; Müller et al., 1999) and tactile (Chung et al., 2002) stimuli. Rather general functions in circuits include automatic gain control (Dayan and Abbott, 2001), network stabilization (Varela et al., 1999), synchronization (Tsodyks et al., 2000), central pattern generation (Manor and Nadim, 2001; Manor et al., 2003), time computation (Loebel and Tsodyks, 2002; Grande and Spain, 2005), response anticipation (Puccini et al., 2007) or termination of UP states (Holcman and Tsodyks, 2006).

It has also been found experimentally that synaptic depression is lesser *in vivo* than *in vitro*, at least in some cortical areas (Sanchez-Vives et al., 1998, 1999; Boudreau and Ferster, 2005). Experimental results suggest that in a constantly active cortical network, the ongoing activity has a modulatory impact on STD (Reig et al., 2006), while the quantity of neurotransmitter released at each synapse increases with disuse (Murthy et al., 2001). Thalamocortical synapses have also been found to be subjected to a chronic state of depression in awake subjects due to high levels of spontaneous firing (Swadlow and Gusev, 2001; Castro-Alamancos and Oldford, 2002; Boudreau and Ferster, 2005; Swadlow et al., 2005). According to recent experiments (Reig et al., 2006), the reduction on STD is proportional to the intensity and duration of the preceding rhythmic activity. In order to do a systematic exploration of the effect that network activity has on STD, we used a biophysical model of the cerebral cortex and we modulated the activity with two different protocols (varying either potassium reversal potentials or specific maximal ionic conductances). In it, we studied the impact that different levels (silent vs. active) and patterns of activity (UP vs. DOWN) have on STD.

We next took advantage of the network model to tackle the inverse question, an experiment only doable *in silico* and leading to interesting predictions; namely, how does synaptic depression influence the emergent activity patterns and the input/output relationships processed in the network? We quantitatively explored the effects that parametrical variation of STD has on the emergent network activity patterns and their possible underlying mechanisms.

## Materials and methods

### Biophysical model

In this paper, we aim not only to reproduce the synaptic depression observed experimentally by Reig et al. (2006), but also to examine the impact of the oscillatory network activity on the mechanisms of synaptic transmission and short-term plasticity and to make predictions (currently not testable experimentally) by exploring the effect that short-term depression at the cell level has onto the whole network. This variety of goals conditions the choice of the model in the sense that it has to: (1) be a validated biologically realistic model containing biophysical elements comparable to those of the experiments; (2) exhibit, at the network level, a similar activity than that of experiments; (3) generate a robust activity under parameter variations; and, (4) include the type of ionic currents that influence synaptic plasticity. According to these requirements, we adapted the biophysical model used in Compte et al. (2003), which was designed to reproduce slow oscillatory activity *in vitro*. In particular, this network model enabled us to analyze different patterns of activity and the effect that each of them has on the STD. We focused then our study on: (1) the STD observed in a silent network versus an active one, (2) UP vs. DOWN states, and finally, (3) the effect that the degree of STD has on the network activity.

The model consists of a population of 1024 pyramidal neurons (excitatory) with two compartments, one for the soma and one for the dendrites (Pinsky and Rinzel, 1994) (each containing specific ion currents found in cortical pyramidal cells), and a population of 256 interneurons (inhibitory) with only one compartment and the basic spiking currents. Both populations are connected to form a network of 1280 neurons of the cerebral cortex arranged on a line. The length of the network model is assumed to be 5 mm of the visual cortex.

Assuming neurons to be electrotonically compact (point neurons), each pyramidal neuron is modeled by a system of two compartments (somatic and dendrite). The somatic compartment (*V*_*s*_) contains the classical spiking currents *I*_*Na*_ and *I*_*K*_, a leak current *I*_*L*_, a fast A-type *K*^+^ current *I*_*A*_, a non-inactivating slow *K*^+^ current *I*_*KS*_, and a *Na*^+^-dependent *K*^+^ current *I*_*KNa*_. The dendrite compartment (*V*_*d*_) contains a high-threshold *Ca*^2+^ current *I*_*Ca*_, a *Ca*^2+^-dependent *K*^+^ current *I*_*KCa*_, a non-inactivating (persistent) *Na*^+^ current *I*_*NaP*_, and an inward rectifier (activated by hyperpolarization) non-inactivating *K*^+^ current *I*_*AR*_. The somatic and dendrite compartments communicate to each other through an electrical coupling *g*_*sd*_ = 0.175 ± 0.1 μS (randomly varied from cell to cell). Both compartments are connected to the network through synaptic currents *I*_syn,*s*_ for the somatic compartment and *I*_syn,*d*_ for the dendrite compartment. The equations for a pyramidal neuron are

(1) $$\begin{array}{c} C_{m}A_{s}\frac{dV_{s}}{dt}=-A_{s}(I_{L}+I_{Na}+I_{K}+I_{A}+I_{KS}+I_{KNa})-g_{sd}(V_{s}-V_{d})-I_{\text{syn},s},\\ C_{m}A_{d}\frac{dV_{d}}{dt}=-A_{d}(I_{Ca}+I_{KCa}+I_{NaP}+I_{AR})-g_{sd}(V_{d}-V_{s})-I_{\text{syn},d}, \end{array}$$

with membrane capacitance *C*_*m*_ = 1 μF/cm^2^ and the areas for the somatic and dendrite compartments being *A*_*s*_ = 0.015 mm^2^ and *A*_*d*_ = 0.035 mm^2^, respectively.

The mono-compartmental interneurons are modeled with the classical Hodgkin–Huxley spiking currents *I*_*Na*_ and *I*_*K*_, and a leak current *I*_*L*_; its differential equation is given by:

(2) $$C_{m}A_{i}\frac{dV_{i}}{dt}=-A_{i}(I_{L}+I_{Na}+I_{K})-I_{\text{syn},i}$$

with the total neuronal surface being *A*_*i*_ = 0.02 mm^2^.

#### Ion channels dynamics

All ionic channels follow the Hodgkin–Huxley formalism and are modeled following Compte et al. (2003). The detailed ionic currents used in the model can be found in the Appendix; here we will only describe the dynamics of activation of a voltage-gated channel. According to the traditional notation, a gating variable *x* is defined by the first-order kinetic equation

(3) $$\frac{dx}{dt}=\phi [\alpha_{x}(V)(1-x)-\beta_{x}(V)x]=\frac{\phi [x_{\infty }(V)-x]}{\tau_{x}(V)}$$

where ϕ is the temperature factor (ϕ = 1, unless otherwise indicated), *x*_∞_(*V*) = α_*x*_(*V*)/(α_*x*_(*V*) + β_*x*_(*V*)) and τ_x_(*V*) = 1/(α_*x*_(*V*) + β_*x*_(*V*)). Each current has its own gating variables, *x*, and its corresponding functions α_*x*_ and β_*x*_. When the time-scale of an activation gating variable is low enough, the model can be simplified substituting *x* by its steady-state function *x*_∞_(*V*).

#### Synaptic dynamics

We assume that neurons are coupled by chemical synapses and we neglect electrical coupling. When a presynaptic neuron “*j*” fires an action potential, it releases neurotransmitters activating both the synaptic strength variable *s*(*t*), that accounts for the amount of neurotransmitters, and the synaptic depression variable *P*_rel_(*t*), which is related with the probability of releasing a neurotransmitter. These neurotransmitters will bind with the postsynaptic receptors, opening ionic channels and allowing for the inflow of postsynaptic current *I*_syn_(*v*) = ∑_*j*_ *g*_syn,*j*_*s*_*j*_(*t*)*P*_rel,*j*_(*t*)(*v* − *V*_syn_).

The synaptic transmission in the model is mediated by an excitatory AMPA (α = 3.48, τ = 2 ms, *V*_syn_ = 0 mV), an excitatory NMDA (α = 0.5, τ = 100 ms, α_*x*_ = 3.48, τ_*x*_ = 2 ms, and *V*_syn_ = 0 mV), and an inhibitory GABA_A_ (α = 1, τ = 10 ms, *V*_syn_ = −70 mV) synaptic currents. The AMPA and GABA_A_ currents follow the first-order kinetic equation

(4) $$\begin{array}{c} \frac{ds}{dt}=\alpha f(V_{\text{pre}})-\frac{s}{\tau },\;\text{where}\;f(V_{\text{pre}})\\ =\frac{1}{\left[1+\exp(-(V_{\text{pre}}-20)/2)\right]}, \end{array}$$

being *f* a sigmoidal function and *V*_pre_ a value related to the presynaptic voltage. The NMDA transmission follows the second-order kinetics

(5) $$\begin{array}{c} \frac{ds}{dt}=\alpha x(1-s)-\frac{s}{\tau },\\ \frac{dx}{dt}=\alpha_{x}f(V_{\text{pre}})-\frac{x}{\tau_{x}}. \end{array}$$

As in Compte, the Mg^2+^-modulation of NMDA was not included in the model.

The rules of connectivity for excitation and inhibition among pools of neurons/compartments are the following [see Compte et al. (2003)]:

- There is an excitatory interaction from somatic to dendrite compartments through AMPA and NMDA transmission (with conductances *g*^AMPA^_EE_ = 5.4 nS and *g*^NMDA^_EE_ = 0.9 nS).
- The somatic compartment excites the interneurons through AMPA and NMDA transmission (with conductances *g*^AMPA^_EI_ = 2.25 nS and *g*^NMDA^_EI_ = 0.5 nS) while, at the same time, assuming that the activity in the soma is equivalent to axonal activity, interneurons inhibit the somatic compartments of pyramidal neurons through GABA_A_ transmission (with conductance *g*^GABA^_IE_ = 4.15 nS).
- There is inhibition among the interneuron population mediated by GABA_A_ transmission (with conductance *g*^GABA^_II_ = 0.165 nS).
- Dendrite compartments do not receive inhibitory inputs. Instead, they are connected into the network through an electrical coupling with the somatic compartment (*g*_*sd*_ = 0.175± 0.1 μS).

The STD incorporated following a phenomenological model (Tsodyks and Markram, 1997; Tsodyks et al., 1998; Dayan and Abbott, 2001). The probability of a neurotransmitter release, *P*_rel_, follows the dynamics

(6) $$\begin{array}{c} \frac{dP_{\text{rel}}}{dt}=\frac{P_{0}-P_{\text{rel}}}{\tau_{\text{rel}}},\\ P_{\text{rel}}(t^{+})\mapsto P_{\text{rel}}(t)f_{D},\text{ if}\;t=t^{k}, \end{array}$$

where *t*^*k*^ is the last spike-time of some presynaptic neuron and *f*_*D*_ is the depression factor (0 ≤ *f*_*D*_ ≤ 1). In other words, every time a presynaptic neuron fires an action potential, the postsynaptic synapse is depressed by a factor *f*_*D*_. When there is no activity (spikes), the depression variable returns to its steady-state *P*_0_ (that is, the synapse recovers the full probability of releasing a neurotransmitter) at a time-rate τ_rel_. In our simulations *f*_*D*_ = 0.9 and τ_rel_ = 400 ms, unless otherwise indicated. We assume that *s*(*t*) and *P*_rel_(*t*) are the same for all synapses of a given presynaptic neuron.

#### Connectivity

The neurons in the network are sparsely connected to each other and equidistantly distributed on a line according to a Gaussian distribution [see Compte et al. (2003)] with zero mean and a standard deviation 125 μm for inhibitory connections and 250 μm for excitatory connections. At the beginning of the simulation, the number of presynaptic connections a neuron receives is fixed to be 20 ± 5 (SD); there can be multiple contacts onto the same target but no autapses are allowed.

#### Numerical methods

The network model was built in C/C++ code (available on request), using a fourth-order Runge–Kutta method with a fixed time step of 0.05 ms for the integration procedure.

### Neurophysiological experimental background

Several studies have reported STD in the cerebral cortex *in vitro* and also *in vivo* (see Introduction for details). In this study, we have been inspired by a recent neurophysiological experiment described in Reig et al. (2006) to explore the effect that network activity has on STD. We will only focus here on the *in vitro* results since those will be the reference for our computational model.

The *in vitro* experiments in Reig et al. (2006) were conducted in cortical slices bathed in an ionic solution called “classical artificial cerebrospinal fluid” (“classical ACSF” in the sequel) which maintains the slice in a silent state (no spontaneous ongoing activity is generated). To induce spontaneous rhythmic activity, the slices were bathed in a “modified ACSF” [“*in vivo*-like ACSF” in the sequel, see Reig and Sanchez-Vives (2007)] that mimics the ionic concentrations *in situ* (Hansen, 1985).

The main difference between the “classical ACSF” and the “*in vivo*-like ACSF” conditions is an increment of extracellular potassium, and a decrement of extracellular calcium and magnesium, see Sanchez-Vives and McCormick (2000) for the exact values. These changes result in an increased excitability of the network. For modeling purposes, we will only take into account the increase in extracellular potassium (see Table 1).

**Table 1:** **Analogies between the *in vitro* and computational experiments**.

*In vitro* experiment	**“Classical ACSF” *KCl* = 2.5 mM**	**“*In vivo*-like ACSF” *KCl* = 3.5 mM**
Computational model	*V*^pyr^_*K*_ = −105 mV	*V*^pyr^_*K*_ = −100 mV
*V*^inh^_*K*_ = −95 mV	*V*^inh^_*K*_ = −90 mV

In order to study the effect that spontaneous network activity had on STD, the authors measured the time-course variation in the amplitude of normalized postsynaptic potentials (PSPs) evoked by external stimulation at 5, 10, and 20 Hz at presynaptic connections. Three different experimental conditions were compared:

1. **Silent state**. Slices were maintained in “classical ACSF” in order to use it as a comparison reference for the STD in the active state.
2. **Silent (ionically modified) state**. Slices were maintained in an “*in vivo*-like ACSF” concentration, prior to the development of organized rhythmic activity. These slices were used to measure the effect that the ionic concentrations in the modified ACSF had, *per se*, on the synaptic depression.
3. **Oscillatory state**. Slices were maintained in an “*in vivo*-like ACSF” concentration in order to study the effect that spontaneous rhythmic network activity had on STD.

#### Computational experiments

We carried out three different computational protocols, aiming at:

**Protocol I.** Reproducing the results obtained in previous neurophysiological experiments by applying periodic stimulation.
**Protocol II.** Exploring the differences of STD between UP and DOWN states by applying stimulation trains.
**Protocol III.** Exploring the impact of STD-modulation on the network activity.

Protocol I consisted in reproducing the experiments described in the previous paragraphs. In our network model, excitatory monosynaptic potentials were activated by applying an external current injection *I*_app_ during 1 ms into a presynaptic pyramidal neuron (monosynaptic connection). The frequencies of activation were designed as in the experimental study (5, 10, and 20 Hz). The amount of external current was calibrated to generate a spike every time the neuron was injected.

Postsynaptic potentials (EPSPs) were measured in a postsynaptic (target) neuron, whose sodium currents (*I*_*Na*_ and *I*_*NaP*_) were blocked in order to prevent the neuron from firing spikes, and thus allowing measurement of the amplitude of postsynaptic potentials (target neuron). The experimental counterpart (Reig et al., 2006) was the inclusion of QX-314, a sodium channel blocker, in the electrode. In our simulations, the target neuron was a specific excitatory pyramidal neuron randomly chosen at the beginning of each simulation. It was in this neuron that all analyses were conducted.

In Protocol II, we then used our computational model and the set up of Protocol I to make predictions about differences between STD in UP and DOWN states. With this aim, trains of five EPSPs were activated during an UP or during a DOWN state at 50 Hz. To determine whether the activation occurred during an UP or a DOWN state, the average synaptic activity of the presynaptic neurons (monosynaptic connections) was evaluated and thresholded. An average synaptic activity over a threshold of −65 mV was considered an UP state (55 spike trains), and a DOWN state (165 spike trains) below a threshold of −70 mV. Intermediate values were considered as transitions between UP and DOWN states, and were neglected from the analysis. We finally measured the ratio between the averaged EPSPs during DOWN states and the averaged EPSPs during UP states.

As the last experiment, Protocol III was designed to make predictions about the impact of STD on the network dynamics. Thus, we were more concerned about making measurements related to the amount of network activity and its propagation. In order to quantify it, we computed a discrete adaptation of wave propagation speed: for every value of the depression factor *f*_*D*_, we randomly chose one of the UP states and estimated the time it took for every neuron to fire the first spike (every neuron has to fire at least one spike); we took the average over different trials and plotted it into a histogram. For the non-oscillatory regime (low *f*_*D*_ values), we took an arbitrary time to be the initial time, since in this regime there is no real wave propagation; in this case, the histogram reflected phase distribution rather than a wave front. From each histogram, we then computed two different indices (*I*_dist_ and *I*_connect_) for the activity propagation. To obtain *I*_dist_, we counted the time it took for all neurons to fire at least one spike (*t*_dist_) from the beginning of an UP state (average of many trials), assuming the wave traveled a distance of 5 mm, *I*_dist_ = 5/*t*_dist_. This index entails a major assumption, namely that the neurons/spikes are ordered; although we knew this was not true for the real wave speed, it worked fine as a comparison index between levels of depression.

The *I*_connect_ index is more realistic, at least from an experimental point of view. Our network model assumes that neurons are numbered on a straight line and that a patch of connectivity for an excitatory (inhibitory) neuron is 0.5 mm (0.25 mm) with an average of 20 postsynaptic contacts. We assumed that the distance between a neuron and its postsynaptic neuron would be *d* = (connectivity patch)/20 × unsigned distance between neurons. At the beginning of an UP state, we took the first 10 different neurons to fire a spike, and we counted the time, *t*_conn_, spent by each of their postsynaptic neurons (monosynapses assumed) to fire a spike (only forward direction in time was allowed). Taking the average over many trials we calculated *I*_connect_ = *d*/*t*_conn_.

## Results

The experimental protocols stated above allowed us to explore, first, how synaptic depression was modulated by the occurrence of oscillatory activity in the network and its mechanistic aspects. Next, we compared synaptic depression in different states (UP and DOWN) of cortical activity and made some predictions in that regard. In the third section, we explored how different levels of STD impacted the emerging rhythmic pattern of cortical network activity and analyzed the cellular and network mechanisms underlying the influence of STD on network activity.

Prior to proceed with the different protocols, we adjusted the network to replicate the experimental conditions of Reig et al. (2006). Under the parameters stated in the Appendix, in the case of no synaptic depression [depression factor *f*_*D*_ = 1, as in Compte et al. (2003)], and without current injection, the network is already in an active state. Average frequency in an UP state is 10 Hz for the excitatory population and 20 Hz for the inhibitory population. The inhibitory UP state is longer so that it can prevent excitatory neurons from recruitment and firing new spikes. This activity will be identified with the oscillatory state (“*in vivo*-like ACSF”) of the experimental conditions (Reig et al., 2006).

### Synaptic depression modulation by network activity

In this section we compared the STD for different levels of activity (from silent to oscillatory) in a cortical network. Aiming to prove that the ultimate mechanism for the modulation of STD was the modulation of the activity, independently of the mechanisms that generate it, we induced the levels of activity in several ways. In this paper we only present two strategies: (1) modifying *K*^+^ reversal potential and (2) modifying the *g*_*KNa*_ conductance. Monosynaptic EPSPs were induced by activating a presynaptic neuron at 5, 10, and 20 Hz (see Protocol I in Materials and Methods). The depression factor was *f*_*D*_ = 0.9 with a recovery rate-time τ_rel_ = 400 ms.

A control EPSP was obtained for each state of the network (see below) by stimulating a presynaptic neuron every second (1 Hz) and determining the amplitude of this EPSP on the target neuron. Note that no LTD (long-term synaptic depression) was included in the model. The control EPSP was calculated as the average over a 25 s simulation and over different trials; its amplitude decreased with the activity in the network. The amplitudes of the postsynaptic potentials (EPSPs) evoked by higher frequencies of stimulation (5, 10, and 20 Hz) were then normalized to the control EPSP value.

#### Modulation of network activity by changing *K*^+^ reversal potential

**Figure 1:**
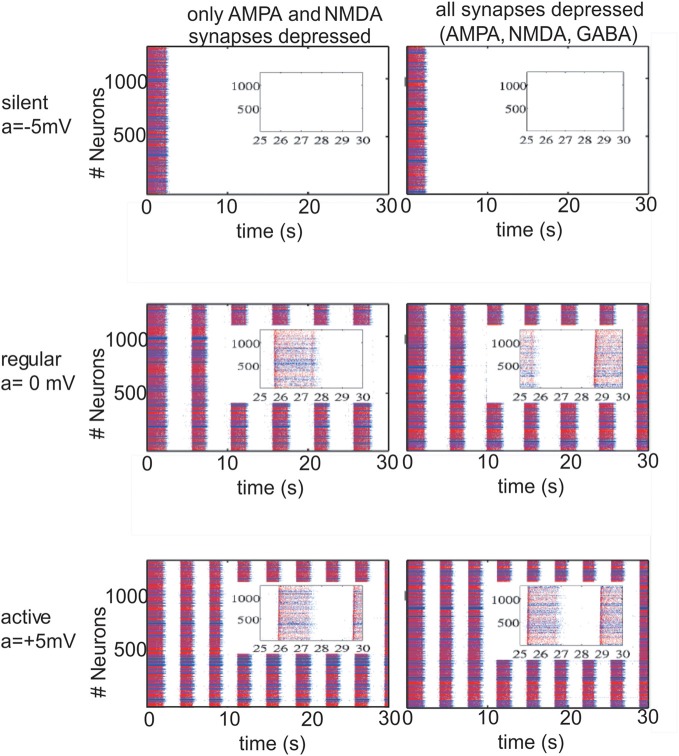
**Different patterns of network activity given by different potassium reversal potential values (*V*^pyr^_*K*_ = −100 + *a* mV, *V*^inh^_*K*_ = −90 + *a* mV).** *Left column*, excitatory synaptic connections depressed (*f*^AMPA,NMDA^_D_ = 0.9); *right column*, excitatory and inhibitory synaptic connections depressed (*f*^AMPA,NMDA,GABA_A_^_D_ = 0.9). Inset figures show the last 5 s of each simulation, respectively. Red (blue) dots correspond to excitatory (inhibitory) neurons, with their actual position in the model network. Upper row panels represent a silent network after the transient period with *V*^pyr^_*K*_ = −105 mV, *V*^inh^_*K*_ = −95 mV. The middle row panels represent a regular network activity, *V*^pyr^_*K*_ = −100 mV, *V*^inh^_*K*_ = −90 mV, slow oscillation frequency of 0.2 Hz and a firing frequency during UP states of 17 Hz (left panel) and 18 Hz (right panel). Lower row represents a more active network as a result of changing *V*^pyr^_*K*_ = −95 mV and *V*^inh^_*K*_ = −85 mV; slow oscillatory activity increases to 0.3 Hz with a firing frequency during UP states of 23 Hz and 24 Hz (left and right panel).

The changes in the ACSF composition in Sanchez-Vives and McCormick (2000) were aimed at changing the excitability of the network. In our model we first alter this excitability by setting the potassium reversal potential at *V*_*k*_ + *a* and choosing *a* in order to achieve different patterns of activity in the network. With *a* = −5 mV (*V*^pyr^_*K*_ = −105 mV, *V*^inh^_*K*_ = −95 mV), we manage to induce a silent network (Figure 1; upper row); with *a* = 0 (*V*^pyr^_*K*_ = −100 mV, *V*^inh^_*K*_ = −90 mV) we return to the slow oscillatory (“*in vivo*-like ACSF”) network with an average frequency during the UP state of 17 Hz for a network with excitatory connections depressed and 18 Hz for a network with excitatory and inhibitory connections depressed (Figure 1; middle row, left and right panels, respectively). To study the effect that an oscillatory network has on STD, rhythmic activity in the network was increased by setting *a* = +5 mV (*V*^pyr^_*K*_ = −95 mV, *V*^inh^_*K*_ = −85 mV) which is indeed more intense, having an UP state average frequency of 23 Hz/24 Hz for a network with excitatory/excitatory and inhibitory connections depressed (Figure 1; lower row, left and right panels, respectively). From Nernst equation, it turns out that *a* = −5 mV, *a* = 0 mV, *a* = 5 mV correspond to concentrations of [*K*]_out_ = 2.88 mM, [*K*]_out_ = 3.5 mM and [*K*]_out_ = 4.2 mM, respectively.

Network activity was exquisitely sensitive to changes in the *K*^+^ reversal potential (Table 1 and Figure 1). Thus, a difference of ±5 mV could bring the network from silence (−105 mV) to 0.3 Hz UP states (−95 mV). Five mV difference (−100 mV and −95 mV) increased both the frequency of occurrence of UP states from 0.2 Hz to 0.3 Hz and the average firing frequency during UP states from 17 Hz to 23 Hz, respectively (Figure 1). This manipulation was an *in computo* replica of the different activity levels achieved experimentally by changes in the ionic concentrations of the ACSF, or by different brain states while *in vivo*.

**Figure 2:**
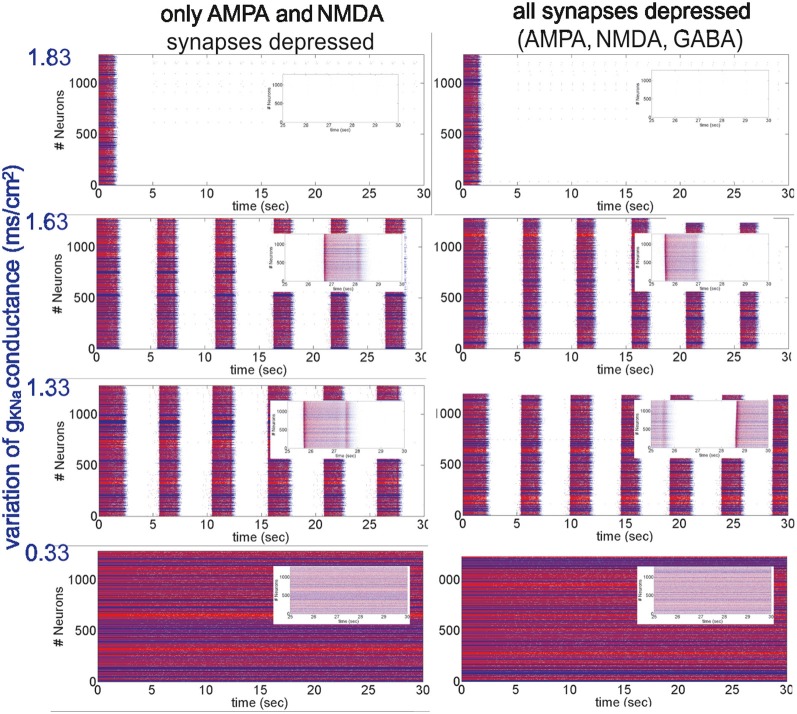
**Different patterns of network activity given by different *Na*^+^-dependent *K*^+^ conductances *g*_*KNa*_.** *Left column*, excitatory synaptic connections depressed (*f*^AMPA,NMDA^_D_ = 0.9); *right column*, excitatory and inhibitory synaptic connections depressed (*f*^AMPA,NMDA,GABA_A_^_D_ = 0.9). Inset figures show the last 5 s of each simulation, respectively. From top to bottom, low activity to high activity with firing frequencies during UP states for the left (right) panel of 0(0) Hz, 12(11) Hz, 17(18) Hz and 30(30) Hz, respectively.

Interestingly, while including STD in excitatory neurons was found to affect the emergent pattern of activity, the inclusion or not of STD in inhibitory synaptic connections did not result in obvious differences neither in the frequency of slow rhythmic activity (compare left panels with right panels in Figures 1 and 2) nor in the firing frequency during UP states.

**Figure 3:**
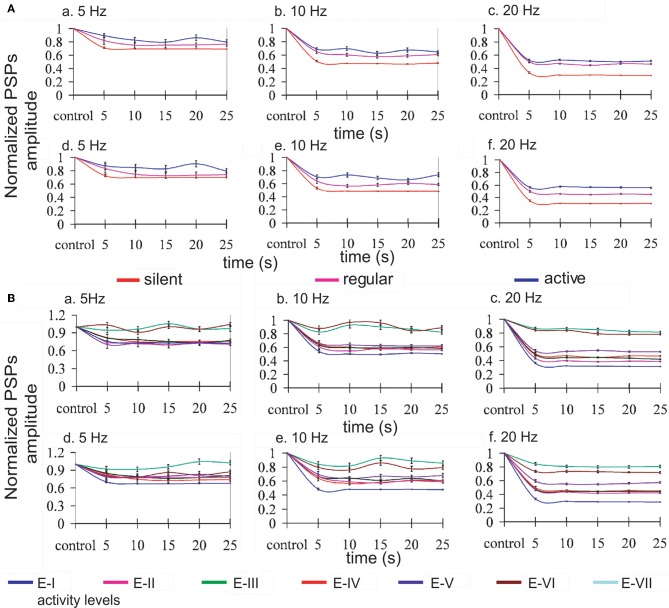
**Modulation of synaptic depression by network activity.** Normalized EPSPs amplitudes evoked by regular current injection. **(A)** Three different levels of activity by changing the *K*^+^ reversal potential: silent network (red line), regular network activity (pink line), and more active network (blue line); and, **(B)** seven different levels of activity by changing the *g*_*KNa*_ conductance from E-I (1.83 mS/cm^2^) to E-VII (0.26 mS/cm^2^) (reference line colors at the bottom of the figure). *Panels a, b, c* represent the normalized PSPs amplitudes when only excitatory synaptic connections were depressed, and *panels d, e, f* when excitatory and inhibitory connections were depressed at the stimulation frequencies of 5, 10, and 20 Hz, respectively. Lines were plotted using cubic splines. At all the stimulation frequencies there is less STD while the network is active than with low or none activity. Average standard error: 2.0% at 5 Hz, 1.5% at 10 Hz, and 0.9% at 20 Hz.

Repetitive activation of monosynaptic connections resulted in a STD, which was contingent to the level of activity in the network (see Figure 3, panel A). Both time course and steady-state amplitude of postsynaptic potentials successfully reproduced the ones obtained experimentally.

#### Modulation of network activity by changing *g*_*KNa*_ conductance

Even though the previous computational results were in agreement with the experimental findings, we explored other forms of variation of network activity levels to ensure that the ultimate mechanism modulating STD was the activity *per se*.

Network activity was then modified in a more dynamic way by changing the conductance of *I*_*KNa*_, *g*_*KNa*_ (Figure 2). In the model used here, the *I*_*KNa*_ current is directly related with the generation and duration of the DOWN states (Compte et al., 2003). As stated in the Appendix, *g*_*KNa*_ was established as 1.33 mS/cm^2^. When the conductance was increased (>40%), it tended to maintain the network in the DOWN state, preventing neurons from overcoming a threshold value and thus keeping the network into a silent state (Figure 2, top panels). Decreasing *g*_*KNa*_ produced the opposite situation: UP states were more easily generated and DOWN states were shorter. Below a certain value (<0.25%) the neurons fired continuously (Figure 2, bottom panels). More precisely, we analyzed STD in seven (E-I to E-VII) artificial cortical networks with different levels of excitability (Figure 2 illustrates only four of them, the other three correspond to intermediate values of *g*_*KNa*_ presenting an intermediate network activity, see below). We took as reference activity the rhythmic activity observed for *g*_*KNa*_ = 1.33 mS/cm^2^ (E-IV), with a firing frequency during UP states of 17 Hz for a network with only excitatory synaptic connections depressed, and 18 Hz for a network with all (excitatory and inhibitory) synaptic connections depressed. Increasing the conductance values to *g*_*KNa*_ = 1.53 mS/cm^2^ and *g*_*KNa*_ = 1.63 mS/cm^2^ (E-III and E-II, respectively), we observed a decrease in the occurrence of UP states and in the firing frequencies during UP states to 14/14 Hz and 12/11 Hz, respectively, for a network with excitatory/all synaptic connections depressed. Increasing further the conductance (for instance, *g*_*KNa*_ = 1.83 mS/cm^2^), the network became silent (E-I). The other way around, decreasing the conductance value to *g*_*KNa*_ = 0.83 mS/cm^2^ (E-V), increased the frequency of rhythmic oscillations, and the firing frequency during UP states to 21/21 Hz, respectively. When decreasing even more the conductance value to *g*_*KNa*_ = 0.33 mS/cm^2^ and *g*_*KNa*_ = 0.26 mS/cm^2^ (E-VI and E-VII, respectively), rhythmic oscillations disappeared and a non-oscillatory pattern appeared with a frequency of 30/30 Hz and 32/31 Hz, respectively, for a network with excitatory/all synaptic connections depressed, and for both *g*_*KNa*_ values. In all of these excitable networks, depression of excitatory synaptic potentials (AMPA and NMDA; Figure 2, left hand panels), and of both, excitatory and inhibitory synaptic potentials (AMPA, NMDA, and GABA_A_; Figure 2, right hand panels) were compared.

As described above for the modulation of network activity by *K*^+^ reversal potential, no significant differences in the pattern of oscillatory activity were observed by including synaptic depression in the inhibitory synaptic potentials. This is also an illustration of the robustness of the effect of activity on synaptic depression. In both cases we found that synaptic depression at the end of the repetitive synaptic stimulation is lower when there is more activity in the network (*V*^pyr^_*K*_ = −95 mV and *V*^int^_*K*_ = −85 mV or *g*_*KNa*_ > 1.33 mS/cm^2^), and is higher when there is less or no activity in the network (*V*^pyr^_*K*_ = −105 mV and *V*^int^_*K*_ = −95 mV or *g*_*KNa*_ < 1.33 mS/cm^2^). The exact quantification is illustrated in Figure 3 for the presynaptic stimulation frequencies of 5, 10, and 20 Hz; average standard errors are of 2.0% at 5 Hz, 1.5% at 10 Hz, and 0.9% at 20 Hz.

Mechanistically, it is important to note that the absolute amplitude of the EPSP (average of amplitudes of the EPSPs) is smaller while the activity in the network increases, and that this difference is reduced as a function of the frequency of the stimulation, being 25, 15, and 1% smaller at 5, 10, and 20 Hz respectively for the modification of the *K*^+^ reversal potential, and 59, 48, and 21% smaller at 5, 10, and 20 Hz respectively for the modification of *g*_*KNa*_ conductance.

**Figure 4:**
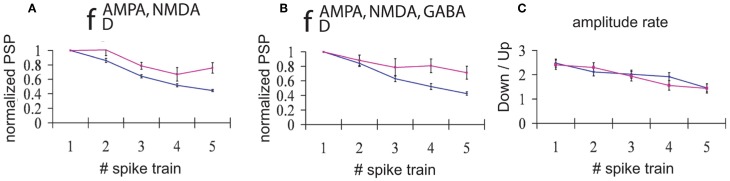
**Input/Output relationships during UP versus DOWN states.** Normalized PSPs amplitude during an *UP state* (pink line) or during a *DOWN state* (blue line). **(A)** Network with only excitatory neurons depressed and **(B)** Network with all connections (excitatory and inhibitory) depressed. An average of 165 trains of 5 spikes at 50 Hz during a DOWN state and 55 trains during an UP state shows that, for all the five spikes in the train, the normalized evoked PSP amplitude is higher during an UP state than during a DOWN state (average standard error = 5.1%(8.2%) during a DOWN state for A and B, respectively, and 6.9%(8.7%) during an UP state for A and B, respectively). **(C)** Proportion between the average of amplitudes of the EPSPs during DOWN state compared with the average of amplitudes of the PSPs during UP state. Blue line: network with only excitatory synaptic depression; pink line: network with synaptic depression in all connections.

In the experimental data from Reig et al. (2006), synaptic depression decreased with cortical ongoing activity while it increased whenever the cortical network was silent. This relationship was true both for cortical slices [Figures 2 and 3 in Reig et al. (2006)] and for *in vivo* recordings [Figures 4 and 5 in Reig et al. (2006)]. Results from the cortical slices *in vitro* are more amenable to compare with our model simulations both because they are an isolated cortical network and because the frequencies of presynaptic stimulation used have been the same (5, 10, and 20 Hz). In the model, STD also decreased with activity in the network, both when activity was regulated by the potassium reversal levels (Figure 3A) or by changes in the *I*_*KNa*_ conductance. Not only in the experiments but also in the model, repetitive stimulation of the presynaptic input was carried out for at least 20 s and the magnitude of the STD was similar. In particular, in silent slices, 20 s of presynaptic stimulation at 5 Hz induced a PSP decay to 70% of the control value, almost identical to the one in the model. In the presence of activity in the network, both in the slices and in the model, STD became negligible. For a frequency of stimulation of 10 Hz and 20 Hz, experimental STD in silent slices decreased synaptic potentials to 50% and 30%, respectively, virtually the same values to the ones in the model. In the presence of rhythmic activity, the synaptic potentials only decayed to 80% and 45%, respectively. This “recovery” of synaptic depression was within the range of the values reached in the model. For higher levels of network activity, STD could be decreased even more, in particular when the *g*_*KNa*_ was reduced (Figure 3B).

From this first section, therefore, we first conclude that global activity in the network can be robustly modulated by small changes in ionic conductances. Second, when the network is silent, synaptic depression is up-regulated, while the activity in the network decreases it. This finding suggests that information transmission in an active network is more secure and repetitive; and high frequency stimuli can be transmitted reliably and without strong decays.

### Synaptic depression in up versus down states

The results presented above demonstrated that ongoing activity in the network decreases STD during spontaneous activity in the model network, in agreement with the experimental observations (Reig et al., 2006; Reig and Sanchez-Vives, 2007). To reinforce these results, a natural question was to explore, in the model, whether there was a difference between transmission during UP and DOWN states. As explained in Protocol II (see Materials and Methods), presynaptic action potential firing (5 spikes at 50 Hz) was induced in the model network. The interval was 1 s ± 100 ms random variation. On a target—postsynaptic (monosynaptic connection) neuron, randomly chosen at the beginning of each simulation, the amplitude of the evoked EPSPs was determined both during an UP and DOWN state, and normalized to the amplitude of the first EPSP in the train. Those trains in which the five EPSPs did not occur totally, either in a DOWN or in an UP state, were ignored.

All trains of EPSPs occurring during DOWN (UP) states for a period of 30 s were averaged. Normalization with respect to the amplitude of the first EPSP in the train (averaged for each train and each simulation) revealed that STD was consistently larger for trains of EPSPs occurring during DOWN states than during UP states (Figure 4). This result holds true both for the case when only excitatory connections are depressed (AMPA and NMDA, Figure 4A), and when all the synaptic connections are depressed (AMPA, NMDA, and GABA_A_, Figure 4B). This computational result, obtained with average standard errors ranging from 5.1% to 8.7%, is proposed here as a prediction for experimental results, and has implications with respect to the information transmission during UP versus DOWN states that will be discussed below.

An interesting result is obtained if we average the amplitude of EPSPs across all trains and simulations, distinguishing between UP and DOWN states. We found that the amplitude of EPSPs is greater in DOWN states than in UP states (Figure 4C); that is, the amplitude of EPSPs is larger during DOWN states than during UP states. This holds true mainly for the first EPSP in the train, since the larger depression during DOWN states leads to values of the ratio between amplitudes close to 1 by the end of the train. As found in the previous section, there is no difference in the ratio of amplitudes between the fully (AMPA, NMDA, and GABA_A_) depressed network and the network with only excitatory synaptic depression (AMPA and NMDA).

### Impact of synaptic depression on the slow rhythmic patterns

**Figure 5:**
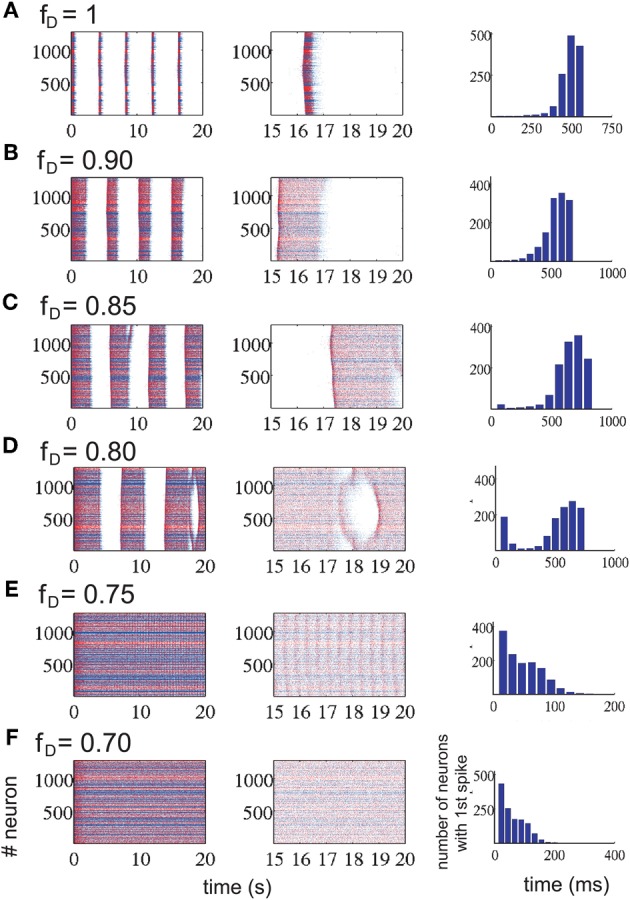
**Network activity modified by the amount of short-term synaptic depression in the excitatory connections (each row represents a different level of STD).** Each row represents a level of depression, from no depression **(A)** to the highest depression factor we have tested **(F)**. Left column represents a 20 s simulation for each depression value; middle column, last 5 s of each of the corresponding simulations. Last column, histogram for the me it takes for all neurons to fire the first spike at the beginning of an UP state (randomly chosen). Increasing synaptic depression increases the duration of the UP states while decreasing the duration of the DOWN states. At some level of synaptic depression, between 0.75 ≤ *f*_*D*_ ≤ 0.85 (from row **D** to row **E**), the network activity changes drastically from an alternating UP state / DOWN state regime to a non-oscillatory regime. The time it takes for all neurons to fire one spike is faster during this last regime.

So far we have shown that network activity has an impact on synaptic depression, but our next goal was the reciprocal problem: does synaptic depression have a remarkable effect on the emergent activity? This finding was achieved by investigating the patterns of emergent activity in a cortical network with different levels of synaptic depression, see Protocol III in Materials and Methods. STD was varied by modifying the depression factor *f*_*D*_ in the model (Dayan and Abbott, 2001). Increasing synaptic depression by decreasing the depression factor *f*_*D*_ induced longer UP states, while DOWN states became shorter (Figure 5). Consistently across trials, there always exist a f^*^_D_ ∈ (0.75, 0.85) where the network activity exhibits a bifurcation in which rhythmic oscillations disappeared, and neurons entered into a non-oscillatory regime for *f*_*D*_ < f^*^_D_. We segregate our analysis in these two different regimes.

Not only the amplitude and frequency of the oscillations were affected by changes in the factor *f*_*D*_, but the propagation of activity in the network was affected too. For a moderate level of depression (*f*_*D*_ ≥ 0.85), the number of neurons recruited grew exponentially in time, while in the non-oscillatory regime or high levels of depression (*f*_*D*_ ≤ 0.75), the shape of the curve exponentially decreased and the time it took for all neurons to fire at least one spike was 4–5 times faster than in the oscillatory regime (see last column of Figure 5). For intermediate levels of depression (0.75 ≤ *f*_*D*_ ≤ 0.85), the effect was intermediate as well: the histogram is *U*-shaped. In this parameter region, the network undergoes the bifurcation from the rhythmic oscillations to the non-oscillatory state.

**Figure 6:**
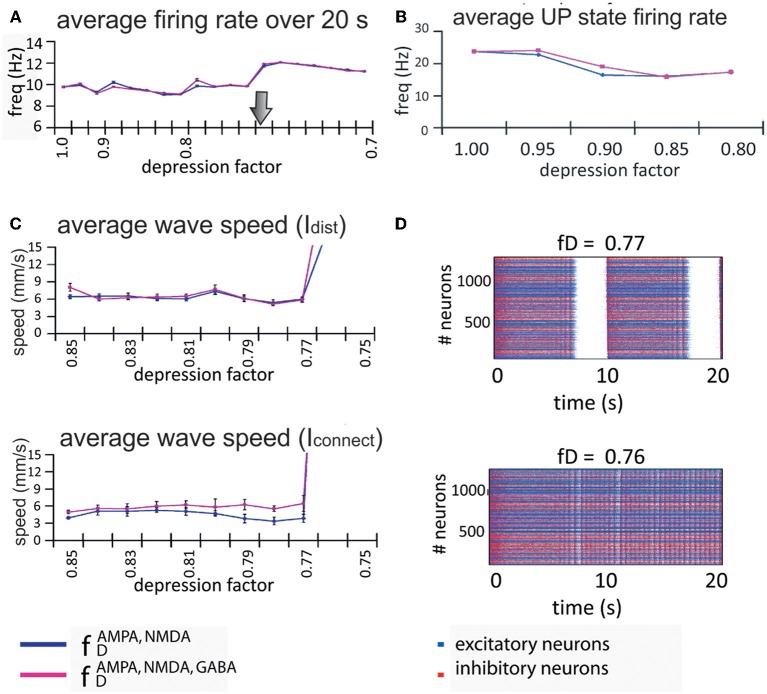
**Firing rate and propagation of UP states in the model network. (A)** Rate of wave propagation in the network is basically maintained during the *UP/DOWN states* regime; this rate is increased when the network activity changes into a continuous firing regime. We found no difference between a network with only excitatory connections depressed (blue line) and a network with both excitatory and inhibitory connections depressed (pink line). The arrow marks the transition point from the oscillatory regime to the continuous firing regime. **(B)** Intra-up-state rate (y-axis) for each depression factor (x-axis). **(C)** Both indices (*I*_dist_, middle panel, and *I*_connect_, bottom panel at the left) show that for values of the depression factor (x-axis) greater than *f*_*D*_ = 0.75, the wave propagation speed (*y*-axis) is more or less maintained at the same level, after which the network passes through some kind of bifurcation and starts increasing its wave propagation speed. Since there are no waves during non-oscillatory regime, the index values are no longer useful. **(D)** Raster plots for two depression factors (middle and bottom panels at the right): at *f*_*D*_ = 0.77 the network activity presents UP and DOWN states while at *f*_*D*_ = 0.76 the network is at a non-oscillatory regime.

We found that the firing rate of the network over a 20-s simulation is basically maintained in each regime (see Figure 6A), being on average 9.6 Hz for the oscillatory regime and 11.6 Hz for the non-oscillatory regime. As for the wave propagation speed, during the oscillatory regime the propagation is basically constant (see Figure 6C). In order to draw conclusions, we analyzed the variation of the wave speed. For a network with this complexity, the standard way to compute the wave propagation speed becomes difficult to apply; thus we approached this problem with two different indices (*I*_dist_ and *I*_connect_) that provide information about the real wave speed.

Despite the non-existence of propagation in an UP state during the non-oscillatory regime, the two propagation indices are still computable, and we used them to detect bifurcation values. During the oscillatory regime, the wave propagation speed is maintained until it reaches the bifurcation where the calculated indices show a drastic increase in the propagation of the wave (Figure 6C). Although it is not shown in the figure, during the non-oscillatory regime our indices exhibited a wave propagation speed between three and four times faster than in the oscillatory regime. It is worth noting that the wave propagation speed is faster when the network has all connections depressed (Figure 6C; pink line) than when only excitatory connections are depressed (Figure 6C; blue line). As mentioned above, it is interesting to observe that the rate and wave propagation speed present basically no changes during the oscillatory regime; the change happens only after the so-called bifurcation.

In order to perform a numerical study of the bifurcation between *f*_*D*_ = 0.85 and *f*_*D*_ = 0.75, we took *f*_*D*_ as our bifurcation parameter, and ran many simulations of the network activity between these values. We found that the network activity changes drastically from an oscillatory regime to a non-oscillatory regime between *f*_*D*_ = 0.77 and *f*_*D*_ = 0.76 (see Figure 6D). Due to the randomness of some of the model parameters, the bifurcation point changes across simulations. For values of *f*_*D*_ higher than the bifurcation value, we can eventually observe how the strips in the raster plots (UP states) start to coalesce before they fuse in a “single strip” of activity after the bifurcation, see for instance Figures 5 and 6D. We have also checked that, even with a fixed connectivity, there are still slight variations of the bifurcation point, due to other random effects (such as initial conditions, for instance).

**Figure 7:**
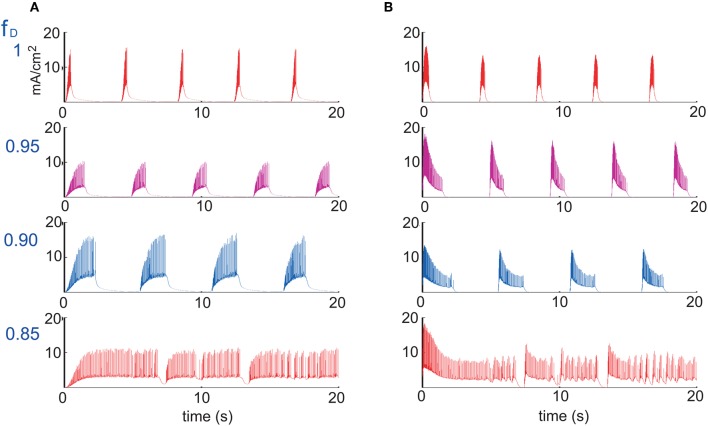
**Currents responsible for the duration of an UP state. (A)** *I*_*KNa*_ current and **(B)** *I*_*KCa*_ current, for four different values of the depression factor *f*_*D*_ (figure shows the case where only excitatory synaptic connections are depressed). *I*_*KNa*_ current increases slower while *I*_*KCa*_ decreases slower during burst as a function of the level of synaptic depression, which tends to lengthen the duration of UP states.

Figure 5 also shows that UP states become longer as synaptic depression increases until they collapse into a non-oscillatory regime at around *f*_*D*_ = 0.76. The rate and wave propagation speed also support this last statement since they also change drastically between 0.76 ≤ *f*_*D*_ ≤ 0.77 (Figures 6A and C). Interestingly, as Figure 6A shows, while *f*_*D*_ decreases (depression increases), the total firing rate remains practically constant. At the same time, we see that intra-UP-state rates decrease as depression increases (Figure 6B). A plausible explanation for this is that synaptic depression reduces the probability of release of neurotransmitters resulting in a decrease of the intra-UP-state rate; on the other hand, the currents responsible for the termination of the UP states (like *I*_*KNa*_ and *I*_*KCa*_ currents) increase/decrease more slowly as synaptic depression gets stronger and so, they contribute to lengthen the UP state interval (Figure 7). The two effects counterbalance to give this apparently constant total rate.

### Cellular mechanisms underlying changes in network dynamics with STD

So far we have described how the increase or decrease of STD has an impact on the network oscillatory dynamics, which can go from a non-oscillatory state (for high values of STD) to an oscillatory state of progressively shorter UP states (for low values of STD). These changes in oscillatory dynamics emerge from the combination of mechanisms integrated in the neurons and their functional connectivity. In order to explore in detail some of these underlying mechanisms, we studied the behavior of the ionic currents in excitatory and inhibitory neurons during network activity.

**Figure 8:**
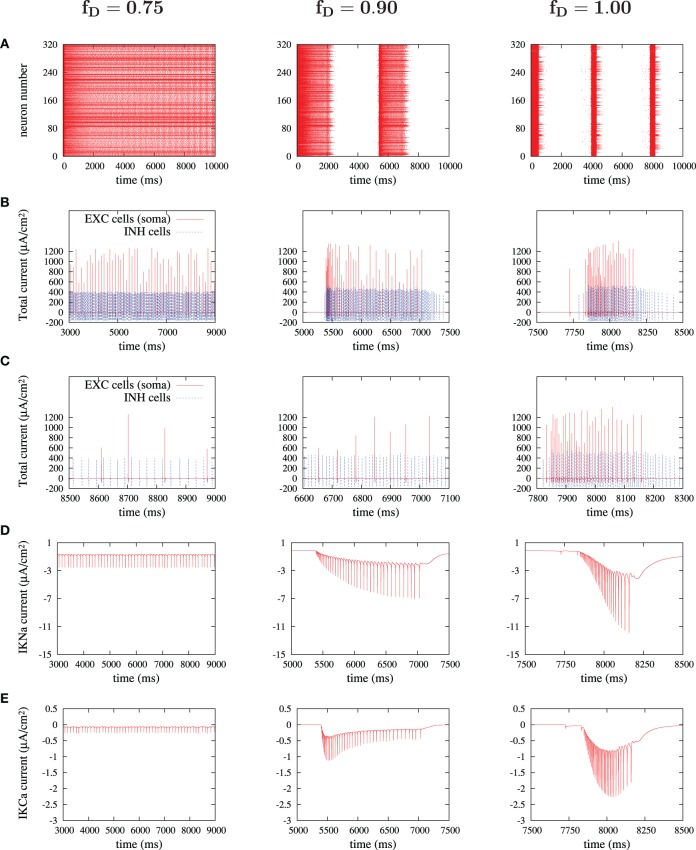
**Currents' dissection during UP states.** For three significant depression levels: **(A)** raster plot during the first 10 seconds; **(B)** sum of currents of an excitatory cell (only somatic compartment shown, in red) and an inhibitory cell (in blue) during an UP state; **(C)** zoom of **(B)** in a 500 ms-window; **(D,E)** sodium-activated and calcium-activated adaptation *K*^+^-currents, *I*_*KNa*_ and *I*_*KCa*_ respectively, in a chosen excitatory cell during an UP state.

The activity of ionic currents was extracted from excitatory and inhibitory neurons during different dynamic states of the simulated cortical network (Figure 8). Special attention was paid to *K*^+^ currents (sodium-dependent and calcium-dependent potassium currents), since they are critical for the termination of UP states (Compte et al., 2003). Figure 8 illustrates from left to right the main findings regarding the behavior of these *K*^+^ currents in three different network states: high, medium and low values of synaptic depression. In Figure 8, we display the results for *f*_*D*_ values of 0.75, 0.90, and 1, although intermediate values were as well checked. Higher values of synaptic depression (as for *f*_*D*_ = 0.75) decreased the strength of recurrence and thus prevented neurons from reaching high values of firing rates (Figures 8B,C, left panels). Under these conditions the activity of the network is non-oscillatory (Figure 8A). When the activation of the *K*^+^ currents is explored (Figures 8D,E, left panels), it is observed that *K*^+^ currents are recruited with each action potential, although the low firing rates prevent from any accumulation of these adaptation mechanisms. Weak adaptation mechanisms then allow the network to maintain a continuous firing without the occurrence of silent periods or DOWN states.

If synaptic depression is slightly lowered to an *f*_*D*_ of 0.90 (middle column in Figure 8), then the network achieves, through its synaptic connectivity, higher firing rates, both in excitatory and in inhibitory neurons (Figures 8B,C, middle panels). These higher firing rates are enough to induce an accumulation in the activation of *K*^+^ currents which consequently silence the network, inducing the generation of DOWN states and thus the oscillatory state (Figures 8D,E, middle panels).

When synaptic depression is eliminated (Figure 8, right panels), the phenomenon observed for medium levels of depression becomes more intense: firing rates in excitatory and inhibitory neurons increase, the accumulation of *K*^+^ currents is more prominent and is reached earlier, thus generating shorter UP states and longer DOWN states. A similar evolution has been observed experimentally and in a model when inhibition is progressively removed, thus inducing a progressive increase in the firing rates of excitatory neurons (Sanchez-Vives et al., 2010).

## Discussion

The impact of synaptic activity, a widespread mechanism in cortical emergent activity and, reciprocally, the effect of network spontaneous activity on synaptic depression were the focus of this study. We explored in a cortical model (Compte et al., 2003) the interactions between STD and spontaneous activity organized in slow rhythmic patterns, reminiscent of those that occur during slow wave sleep (Steriade et al., 1993). A mutual interaction was found between synaptic depression and network activity, such that network activity down-regulates synaptic depression, and synaptic depression modulates the patterns of emergent network activity. The first test (Protocol I) carried out in the model was inspired by previous experimental results (Reig et al., 2006), where changes in cortical synaptic depression were quantified against ongoing activity. However, testing in the context of the model has now allowed us (1) to find out the extent of the reproducibility of the experimental results in the model and to carry out a detailed quantification of the effects of activity on synaptic depression, (2) to generalize the effect of different forms of activity over synaptic depression, (3) to estimate the effects on excitatory and inhibitory transmission, as well as the impact of excitatory vs. inhibitory depression on the network, and (4) to determine the effects that parametric changes in STD have on global network emergent activity, modifications difficult to attain experimentally.

### Activity modulation of excitatory and inhibitory synaptic depression

In order to regulate the activity in the artificial cortical network and to explore the consequences on synaptic depression, two different strategies were used: (1) modification of *V*^+^_*K*_ reversal potential, and (2) modification of the sodium-activated *K*^+^ conductance, *g*_*KNa*_, a critical mechanism in Compte et al. (2003) for the termination of UP states. The objective here was not to explore the impact that these changes had on UP states, but to control the global amount of activity, and to analyze the resulting effects on STD. As described experimentally (Reig et al., 2006), STD in the model decreased with the level of activity in the network. The amplitude of EPSPs was lower for higher levels of activity in the network, since they were pre-depressed by the ongoing activity. Repetitive synaptic stimulation induced decay in the EPSPs amplitude that eventually reached a plateau (Figures 3A and B). This plateau had a different value depending on the amount of activity in the network, being lower for a silent or low activity network than in an active one. Similar results have been observed both experimentally by Reig et al. (2006) and in a model (Tsodyks and Markram, 1997). A chronic state of depression of thalamocortical synapses *in vivo* has also been argued (Swadlow and Gusev, 2001; Castro-Alamancos and Oldford, 2002; Boudreau and Ferster, 2005; Swadlow et al., 2005).

We did not find differences in the network modulation of STD between a network with only depressing excitatory connections and one where both excitatory and inhibitory connections were depressed (Figures 3 and 4). This might be due to the total ratio between the number of excitatory and inhibitory connections in our network model (≃4). Experimentally, depression of IPSPs is weaker than that of EPSPs (Galaretta and Hestrin, 1998; Varela et al., 1999; Chelaru and Dragoi, 2008).

Our results suggest that the activity in the network sets the synapses in a different steady-state thus displaying less depression, and transmitting information with lesser or no decay.

### Synaptic transmission and short term depression during up versus down states

The input–output relationship of the network was compared across UP and DOWN states (Protocol II). In our model, synaptic depression was larger during DOWN states than during UP states (Figure 4). Lesser depression during UP states with respect to DOWN states has been described experimentally (Crochet et al., 2005; Reig and Sanchez-Vives, 2007) but only for pair-pulse stimulation and not for longer stimulation series. Longer series of stimulation of thalamocortical connections at similar frequencies were described to induce an augmentation rather than a depression in cortex *in vivo* (Steriade et al., 1998), although not taking into account UP and DOWN states. Our computational results, showing lesser synaptic depression in UP than in DOWN states, should be therefore considered an experimental prediction. The tendency to change the network state (to start or finish an UP state) with repetitive electrical stimulation (Petersen, 2002; Haider et al., 2006; Reig and Sanchez-Vives, 2007) posits though a problem to obtain series of *circa* 5 stimuli occurring only during DOWN states or UP states. Nevertheless, a different degree of synaptic depression during UP and DOWN states is coherent both with the model results presented here, as well as with experimental results showing that STD in a silent network is larger than in an active one (Crochet et al., 2006; Reig et al., 2006; Reig and Sanchez-Vives, 2007). Even at different time scale and with a global amount of activity, a DOWN state represents a silent state of the network, and an UP state, an activated network.

On the other hand, the amplitude of an EPSP evoked by a single action potential was in the model larger at DOWN states with respect to UP states (Figure 4C), as found experimentally (Crochet et al., 2005). The relative amplitude of PSPs in UP versus DOWN states has been a matter of controversy. Responses evoked by the same stimulus have been found to be of lesser amplitude in UP than in DOWN states in certain cortical areas (e.g., barrel cortex) and stimulation conditions (Petersen, 2002; Sachdev et al., 2004; Crochet et al., 2005, 2006; Hasenstaub et al., 2007). A reason argued for this lesser amplitude is the proximity to the reversal potential of glutamate receptor channels at depolarized values, a mechanism that was responsible also for the result in the model. On the other hand, experimental results have also reported an increased output/input during UP states to a stimulus induced either naturally or electrically (Azouz and Gray, 1999; Haider et al., 2007; Reig and Sanchez-Vives, 2007), the higher excitability and responsiveness of the network during UP states being a main argument supporting these results.

Other contributing factors are neuromodulators released during states of arousal, like acetylcholine, which down-regulate synaptic depression (Gil et al., 1997). A lesser depression in UP states and in active networks implies a higher reliability in information transmission during alert states. Furthermore, increased noise during activated states contributes to the responsiveness, signal detection, and timing accuracy of the input/output (Ho and Destexhe, 2000; Wolfart et al., 2005; Marti et al., 2008).

### The effect of synaptic depression on the network emergent activity

Recurrent activity in the network supports the organization of spontaneous activity into UP and DOWN states (Steriade et al., 1993). The liaison between synaptic and intrinsic membrane properties determines the specific emerging activity patterns; however, the exact mechanisms controlling the different parameters of the oscillations remain elusive. One factor proposed in several models as a critical element to regulate the emergent activity or as a mechanism to end UP states is synaptic depression (Bazhenov et al., 2002; Hill and Tononi, 2005; Holcman and Tsodyks, 2006).

Our model allowed us to test the effect on the UP and DOWN states of a gradual increase in STD (Protocol III). This model reproduces slow oscillatory activity generated by the cortex *in vitro* (Sanchez-Vives and McCormick, 2000) without synaptic depression. Here, we introduced STD in the excitatory or in both, excitatory and inhibitory, connections. Against intuitive predictions of network behavior, a gradual increase in STD (decreasing *f*_*D*_) resulted in a progressive elongation of the UP states, and a shortening of the DOWN states. The progression led from a slow oscillatory (<1 Hz) to a non-oscillatory regime (≃10 Hz) after crossing a threshold in which UP states started to merge (see Figure 5). In this intermediate state, most of the neurons fired in a regular way, but some of the neurons were yet engaged in oscillatory sequences. This state of threshold is, to some extent, similar to the one observed by Holcman and Tsodyks (2006) in a mean field model. During these states of bifurcation, not only UP states started to merge, but there was a drastic change in the network's dynamics, including the time to the first spike (Figure 5), the UP states firing rate (Figure 6A), and the propagation in the network (Figure 6C).

A decaying strength in the recurrent connectivity during UP states, due to depression, slightly decreased the resulting firing frequency in the network. This slight decay in the firing rate was enough though to induce a slower accumulation of the *I*_*KNa*_ current, and a slower decrement of the *I*_*KCa*_ current (Figure 7), which in this model were critical to control the duration of UP states and to maintain DOWN states (Compte et al., 2003). Progressively, longer UP and shorter DOWN states resulted in UP states merging into continuous depolarization. Interestingly, while STD increased and the network was still in an oscillatory regime, the rate and speed of propagation showed no modification, providing that the excitatory–inhibitory balance was not modified. This result contrasts with the case described in Bressloff (1999) of an infinite number of globally connected in-phase integrate and fire neurons, in which the rate decreases with depression. However, many features of our model can account for this difference: local connectivity, finite size of the network, presence of inhibitory neurons, different distribution of phases, etc. Links with the study of Mejias et al. (2010) of a rate model with noisy dynamical synapses are also worth mentioning. In Mejias et al. (2010), it is shown that long stays in the up state would occur more frequently for high values of *f*_*D*_. Since we use a detailed biophysical network with no noise added to the dynamical synapses, differences have to be carefully interpreted but the fact that we get longer stays in the up state when decreasing *f*_*D*_ may indicate that either the noise in the dynamical synapses or the detailed channel dynamics in our model could explain these differences. As we understand, our model itself serves as a mechanistic explanation of how using plausible neurobiological assumptions, network activity, and depression levels can interplay, but the complexity of the model still provides too many possible explanations. A deeper study of the interplay of network mechanisms was made by dissecting the behavior of ionic currents in excitatory and inhibitory cells during the different network states, see Figure 8. This exploration revealed more detailed cellular and network mechanisms contributing to the lengthening of UP states and shortening of DOWN states when synaptic depression was increased. Decreasing synaptic depression resulted in higher firing rates of both excitatory and inhibitory neurons. This increase in firing rates results in a more efficient recruitment of sodium-dependent (Figure 8D) and calcium-dependent (Figure 8E) potassium currents, thus preventing neurons from firing and contributing to the further shortening of the UP states.

## Author contributions

All authors have contributed in the different tasks: conceiving, designing and revising the manuscript; and, acquiring, analyzing and interpreting data. All have given the final approval of this version.

### Conflict of interest statement

The authors declare that the research was conducted in the absence of any commercial or financial relationships that could be construed as a potential conflict of interest.

## Appendix

### Ionic currents and parameter values

Ionic currents for somatic and dendrite compartments were taken from Compte et al. (2003). The sodium current, *I*_*Na*_ = *g*_*Na*_ *m*^3^_∞_ *h* (*v* − *V*_*Na*_), has a rapid activation variable replaced by its steady-state *m*_∞_ = α_*m*_/(α_*m*_ + β_*m*_) with α_*m*_ = 0.1 (*v* + 33)/[1 −exp(−(*v* + 33)/10)] and β_*m*_ = 4 exp(−(*v* + 53.7)/12), and an inactivation variable defined from α_*h*_ = 0.07 exp(−(*v* + 50)/10) and β_*h*_ = 1/[1 + exp(−(*v* + 20)/10)]; the maximal conductance is *g*_*Na*_ = 50 mS/cm^2^. The delayed rectifier, *I*_*K*_ = *g*_*K*_*n*^4^(*v* − *V*_*K*_), has an inactivation kinetics driven by α_*n*_ = 0.01 (*v* + 34)/[1 − exp(−(*v* + 34)/10)] and β*n* = 0.125 exp[−(*v* + 44)/25], with a maximal conductance *g*_*K*_ = 10.5 mS/cm^2^. The leakage current, *I*_*L*_ = *g*_*L*_(*v* − *V*_*L*_), has a maximal conductance *g*_*L*_ = 0.0667 ± 0.0067 mS/cm^2^ and is a passive channel. The fast A-type *K*^+^ channel, *I*_*A*_ = *g*_*A*_ *m*^3^_∞_ *h*_∞_ (*v* − *V*_*K*_), has a rapid activation variable replaced by its steady-state *m*_∞_ = 1/[1−exp(−(*v* + 50)/20)] and its inactivation variable by *h*_∞_ = 1/[1+exp((*v* + 80)/6)] with τ_*h*_ = 15 ms; it has a maximal conductance of *g*_*A*_ = 1 mS/cm^2^. The non-inactivating *K*^+^ channel, *I*_KS_ = *g*_*KS*_ *m*_∞_ (*v* − *V*_*K*_), has an activation controlled by *m*_∞_ = 1/[1+exp(−(*v* + 34)/6.5)] with τ_*m*_ = 8/[exp(−(*v* + 55)/30) + exp((*v* + 55)/30)] with a conductance *g*_*KS*_ = 0.576 mS/cm^2^.

The persistent sodium channel, *I*_*NaP*_ = *g*_*NaP*_ *m*^3^_∞_(*v* − *V*_*Na*_), has a conductance *g*_*NaP*_ = 0.0686 mS/cm^2^, and activates according to *m*_∞_ = 1/[1+exp(−(*v* + 55.7)/7.7)]. The inwardly rectifying *K*^+^ channel, *I*_*AR*_ = *g*_*AR*_ *h*_∞_ (*v* − *V*_*K*_), has a conductance *g*_*AR*_ = 0.0257 mS/cm^2^, and activates instantaneously according to *h*_∞_ = 1/[1+exp((*v* + 75)/4)].

The high-threshold *Ca*^2+^-channel, *I*_*Ca*_ = *g*_*Ca*_ *m*^2^_∞_ (*v* − *V*_*Ca*_), has a conductance *g*_*Ca*_ = 0.43 mS/cm^2^, and activates instantaneously at very depolarized voltages according to *m*_∞_ = 1/[1+exp(−(*v* + 20)/9)]. The *Ca*^2+^-dependent *K*^+^ channel, *I*_*KCa*_ = *g*_*KCa*_[*Ca*^2+^]/([*Ca*^2+^] + *K*_*D*_)(*v* − *V*_*K*_), where *K*_*D*_ = 30 μM and *g*_*KCa*_ = 0.57 mS/cm^2^, activates instantaneously in the presence of intracellular calcium [*Ca*^2+^] (Wang, 1998), which follows the first-order kinetics

$$\frac{d[Ca^{2+}]}{dt}=-\alpha_{Ca}A_{d}I_{Ca}-\frac{\left[Ca^{2+}\right]}{\tau_{Ca}},$$

with α_*Ca*_ = 0.005 μM/(nA ms) and τ_*Ca*_ = 150 ms.

The *Na*^2+^-dependent *K*^+^ channel, *I*_*KNa*_ = *g*_*KNa*_*w*([*Na*+])(*v* − *V*_*K*_) depends on the intracellular sodium concentration through *w*([*Na*+]) = 0.37/[1+(38.7/[*Na*^+^])^3.5^] and the following dynamics for the concentration:

$$\begin{array}{c} \frac{d[Na^{+}]}{dt}=-\alpha_{Na}(A_{s}I_{Na}+A_{d}I_{NaP})\\ \;-R_{\text{pump}}\left\{\frac{{\left[Na^{+}\right]}^{3}}{\left({\left[Na^{+}\right]}^{3}+15^{3}\right)}-\frac{{\left[Na^{+}\right]}_{eq}^{3}}{\left({\left[Na^{+}\right]}_{eq}^{3}+15^{3}\right)}\right\}, \end{array}$$

with α_*Na*_ = 0.01 mM/(nA ms), *R*_pump_ = 0.018 mM/ms and [*Na*^+^]_*eq*_ = 9.5 mM.

Reversal leakage potential is *V*_*L*_ = −60.95 mV, sodium reversal potential, *V*_*Na*_ = 55 mV, potassium reversal potential, *V*_*K*_ = −100 mV and calcium reversal potential, *V*_*Ca*_ = 120 mV.

As for the interneurons, the sodium current, *I*_*Na*_ = *g*_*Na*_*m*^3^*h*(*v* − *V*_*Na*_), has an activation variable controlled by α*m* = 0.5(*v* + 35)/[1−exp(−(*v* + 35)/10)] and β_*m*_ = 20 exp(−(*v* + 60)/18), and an inactivation gating variable controlled by α_*h*_ = 0.35 exp(−(*v* + 58)/20) and β_*h*_ = 5/[1+exp(−(*v* + 28)/10)]; it has a maximal conductance of *g*_*Na*_ = 35 mS/cm^2^. The delayed rectifier, *I*_*K*_ = *g*_*K*_ *n*^4^(*v* − *V*_*K*_), has a conductance *g*_*K*_ = 9 mS/cm^2^, and activates according to α*n* = 0.05(*v* + 34)/[1−exp(−(*v* + 34)/10)] and β_*n*_ = 0.625 exp(−(*v* + 44)/80). The leakage current, *I*_*L*_ = *g*_*L*_(*v* − *V*_*L*_), has a maximal conductance *g*_*L*_ = 0.1025 ± 0.0025 mS/cm^2^ and is a passive channel. Finally, leakage reversal potential is *V*_*L*_ = −63.8 mV, sodium reversal potential, *V*_*Na*_ = 55 mV, and potassium reversal potential, *V*_*K*_ = −90 mV.

## References

[B1] Azouz R. Gray C. (1999). Cellular mechanisms contributing to response variability of cortical neurons *in vivo*. J. Neurosci. 19, 2209–2223 10066274 10.1523/JNEUROSCI.19-06-02209.1999 PMC6782570

[B2] Bazhenov M. Timofeev I. Steriade M. Sejnowski T. (2002). Model of thalamocortical slow wave sleep oscillations and transitions to activated states. J. Neurosci. 22, 8691–8704 12351744 10.1523/JNEUROSCI.22-19-08691.2002 PMC6757797

[B3] Borst J. G. G. (2010). The low synaptic release probability *in vivo*. Trends Neurosci. 33, 259–266 10.1016/j.tins.2010.03.003 20371122

[B4] Boudreau C. Ferster D. (2005). Short-term depression in thalamocortical synapses of cat primary visual cortex. J. Neurosci. 25, 7179–7190 10.1523/JNEUROSCI.1445-05.2005 16079400 PMC6725224

[B5] Bressloff P. C. (1999). Mean-field theory of globally coupled integrate-and-fire neural oscillators with dynamic synapses. Phys. Rev. E 60, 2160–2170 11970009 10.1103/physreve.60.2160

[B6] Carandini M. Heeger D. Senn W. (2002). A synaptic explanation of suppression in visual cortex. J. Neurosci. 22, 10053–10065 12427863 10.1523/JNEUROSCI.22-22-10053.2002 PMC6757815

[B7] Castro-Alamancos M. Oldford E. (2002). Cortical sensory suppression during arousal is due to the activity-dependent depression of thalamocortical synapses. J. Physiol. 541, 319–331 10.1113/jphysiol.2002.016857 12015438 PMC2290309

[B8] Chance F. Nelson S. Abbott L. (1998). Synaptic depression and the temporal response characteristics of v1 cells. J. Neurosci. 18, 4785–4799 9614252 10.1523/JNEUROSCI.18-12-04785.1998 PMC6792683

[B9] Chelaru M. Dragoi V. (2008). Asymmetric synaptic depression in cortical networks. Cereb. Cortex 18, 771–788 10.1093/cercor/bhm119 17693394

[B10] Chung S. Li X. Nelson S. (2002). Short-term depression at thalamocortical synapses contributes to rapid adaptation of cortical sensory responses *in vivo*. Neuron 34, 437–446 10.1016/S0896-6273(02)00659-1 11988174

[B11] Compte A. Sanchez-Vives M. McCormick D. Wang X.-J. (2003). Cellular and network mechanisms of slow oscillatory activity (<1 hz) and wave propagations in a cortical network model. J. Neurophysiol. 89, 2707–2725 10.1152/jn.00845.2002 12612051

[B12] Cook D. Schwindt P. Grande L. Spain W. (2003). Synaptic depression in the localization of sound. Nature 421, 66–70 10.1038/nature01248 12511955

[B13] Crochet S. Chauvette S. Boucetta S. Timofeev I. (2005). Modulation of synaptic transmission in neocortex by network activities. Eur. J. Neurosci. 21, 1030–1044 10.1111/j.1460-9568.2005.03932.x 15787708

[B14] Crochet S. Fuentealba P. Cisse Y. Timofeev I. Steriade M. (2006). Synaptic plasticity in local cortical network *in vivo* and its modulation by the level of neuronal activity. Cereb. Cortex 16, 618–631 10.1093/cercor/bhj008 16049189

[B15] Dayan P. Abbott L. (2001). Theoretical neuroscience in Model Neurons I: Neuroelectronics: Synaptic Conductances, Section 5.8 (Cambridge, MA: MIT press), XV–460

[B16] Freeman T. S. Durand Kiper D. Carandini M. (2002). Suppression without inhibition in visual cortex. Neuron 35, 759–771 10.1016/S0896-6273(02)00819-X 12194874

[B17] Galaretta M. Hestrin M. (1998). Frequency-dependent synaptic depression and the balance of excitation and inhibition in the neocortex. Nat. Neurosci. 1, 587–594 10.1038/2882 10196566

[B18] Gil Z. Connors B. Amitai Y. (1997). Differential regulation of neocortical synapses by neuromodulators and activity. Neuron 19, 679–686 10.1016/S0896-6273(00)80380-3 9331357

[B19] Grande L. Spain W. (2005). Synaptic depression as a timing device. Physiology 20, 201–210 10.1152/physiol.00006.2005 15888577

[B20] Haider B. Duque A. Hasenstaub A. McCormick D. (2006). Neocortical network activity *in vivo* is generated through a dynamic balance of excitation and inhibition. J. Neurosci. 26, 4535–4545 10.1523/JNEUROSCI.5297-05.2006 16641233 PMC6674060

[B21] Haider B. Duque A. Hasenstaub A. Yu Y. McCormick D. (2007). Enhancement of visual responsiveness by spontaneous local network activity *in vivo*. J. Neurophysiol. 97, 4186–4202 10.1152/jn.01114.2006 17409168

[B22] Hansen A. (1985). Effect of anoxia on ion distribution in the brain. Physiol. Rev. 65, 101–148 3880896 10.1152/physrev.1985.65.1.101

[B23] Hasenstaub A. Sachdev R. McCormick D. (2007). State changes rapidly modulate cortical neuronal responsiveness. J. Neurosci. 27, 9607–9622 10.1523/JNEUROSCI.2184-07.2007 17804621 PMC6672966

[B24] Hill S. Tononi G. (2005). Modeling sleep and wakefulness in the thalamocortical system. J. Neurophysiol. 93, 1671–1698 10.1152/jn.00915.2004 15537811

[B25] Ho N. Destexhe A. (2000). Synaptic background activity enhances the responsiveness of neocortical pyramidal neurons. J. Neurophysiol. 84, 1488–1496 10980021 10.1152/jn.2000.84.3.1488

[B26] Holcman D. Tsodyks M. (2006). The emergence of up and down states in cortical networks. PLoS Comput. Biol. 2:e23 10.1371/journal.pcbi.0020023 16557293 PMC1409813

[B27] Lauritzen T. Krukowski A. Miller K. (2001). Local correlation-based circuitry can account for responses to multi-grating stimuli in a model of cat v1. J. Neurophysiol. 86, 1803–1815 11600641 10.1152/jn.2001.86.4.1803

[B28] Loebel A. Tsodyks M. (2002). Computation by ensemble synchronization in recurrent networks with synaptic depression. J. Comput. Neurosci. 13, 111–124 10.1023/A:1020110223441 12215725

[B29] Manor Y. Bose A. Booth V. Nadim F. (2003). Contribution of synaptic depression to phase maintenance in a model rhythmic network. J. Neurophysiol. 90, 3513–3528 10.1152/jn.00411.2003 12815020

[B30] Manor Y. Nadim F. (2001). Synaptic depression mediates bistability in neuronal networks with recurrent inhibitory connectivity. J. Neurosci. 21, 9460–9470 11717380 10.1523/JNEUROSCI.21-23-09460.2001 PMC6763901

[B31] Marti D. Deco G. Mattia M. Gigante G. Del Giudice P. (2008). A fluctuation-driven mechanism for slow decision processes in reverberant networks. PLoS ONE 3:e2534 10.1371/journal.pone.0002534 18596965 PMC2432027

[B32] Mejias J. Kappen H. Torres J. (2010). Irregular dynamics in up and down cortical states. PLoS ONE 5:e13651 10.1371/journal.pone.0013651 21079740 PMC2975677

[B33] Müller J. Metha A. Krauskopf J. Lennie P. (1999). Rapid adaptation in visual cortex to the structure of images. Science 285, 1405–1408 10.1126/science.285.5432.1405 10464100

[B34] Murthy V. Schikorski T. Stevens C. Zhu Y. (2001). Inactivity produces increases in neurotransmitter release and synapse size. Neuron 32, 673–682 10.1016/S0896-6273(01)00500-1 11719207

[B35] Nelson S. (1991a). Temporal interactions in the cat visual systems. i. orientation-selective suppression in the visual cortex. J. Neurosci. 11, 344–356 1992005 10.1523/JNEUROSCI.11-02-00344.1991 PMC6575230

[B36] Nelson S. (1991b). Temporal interactions in the cat visual systems. ii. suppressive and facilitatory effects in the lateral geniculate nucleus. J. Neurosci. 11, 357–368 1992006 10.1523/JNEUROSCI.11-02-00357.1991 PMC6575213

[B37] Nelson S. (1991c). Temporal interactions in the cat visual systems. iii. pharmacological studies of cortical suppression suggest a presynaptic mechanism. J. Neurosci. 11, 369–380 1992007 10.1523/JNEUROSCI.11-02-00369.1991 PMC6575226

[B38] Petersen C. (2002). Short-term dynamics of synaptic transmission within the excitatory neuronal network of rat layer 4 barrel cortex. J. Neurophysiol. 87, 2904–2914 12037194 10.1152/jn.2002.87.6.2904

[B39] Petersen C. Hahn T. Mehta M. Grinvald A. Sakmann B. (2003). Interaction of sensory responses with spontaneous depolarization in layer 2/3 barrel cortex. Proc. Natl. Acad. Sci. U.S.A. 100, 13638–13643 10.1073/pnas.2235811100 14595013 PMC263866

[B40] Pinsky P. Rinzel J. (1994). Intrinsic and network rhythmogenesis in a reduced traub model for ca3 neurons. J. Comput. Neurosci. 1, 39–60 8792224 10.1007/BF00962717

[B41] Puccini G. Sanchez-Vives M. Compte A. (2007). Integrated mechanisms of anticipation and rate-of-change computations in cortical circuits. PLoS Comput. Biol. 3:e82 10.1371/journal.pcbi.0030082 17500584 PMC1866356

[B42] Reig R. Gallego R. Nowak L. Sanchez-Vives M. (2006). Impact of cortical network activity on short-term synaptic depression. Cereb. Cortex 16, 688–695 10.1093/cercor/bhj014 16107589

[B43] Reig R. Sanchez-Vives M. (2007). Synaptic transmission and plasticity in an active cortical network. PLoS ONE 2:e670 10.1371/journal.pone.0000670 17668052 PMC1925142

[B44] Sachdev R. Ebner F. Wilson C. (2004). Effect of subthreshold up and down states on the whisker-evoked response in somatosensory cortex. J. Neurophysiol. 92, 3511–3521 10.1152/jn.00347.2004 15254074

[B45] Sanchez-Vives M. McCormick D. (2000). Cellular and network mechanisms of rhythmic recurrent activity in neocortex. Nat. Neurosci. 3, 1027–1034 10.1038/79848 11017176

[B46] Sanchez-Vives M. Nowak L. McCormick D. (1998). Is synaptic depression prevalent *in vitro* and does it contribute to contrast adaptation? Soc. Neurosci. Abstr. 24, 896

[B47] Sanchez-Vives M. Nowak L. McCormick D. (1999). Why might cortical synaptic depression be lesser *in vivo* than *in vitro*? Soc. Neurosci. Abstr. 25, 2191

[B48] Sanchez-Vives M. V. Mattia M. Compte A. Perez-Zabalza M. Winograd M. Descalzo V. F. Reig R. (2010). Inhibitory modulation of cortical up states. J. Neurophysiol. 104, 1314–1324 10.1152/jn.00178.2010 20554835

[B49] Steriade M. Nunez A. Amzica F. (1993). A novel slow (<1 Hz) oscillation of neocortical neurons *in vivo*: depolarizing and hyperpolarizing components. J. Neurosci. 13, 3252–3265 8340806 10.1523/JNEUROSCI.13-08-03252.1993 PMC6576541

[B50] Steriade M. Timofeev I. Grenier F. Durmuller N. (1998). Role of thalamic and cortical neurons in augmenting responses and self-sustained activity: dual intracellular recordings *in vivo*. J. Neurosci. 18, 6425–6443 9698333 10.1523/JNEUROSCI.18-16-06425.1998 PMC6793197

[B51] Stoelzel C. Bereshpolova Y. Gusev A. Swadlow H. (2008). The impact of an lgnd impulse on the awake visual cortex: synaptic dynamics and the sustained/transient distinction. J. Neurosci. 28, 5018–5028 10.1523/JNEUROSCI.4726-07.2008 18463255 PMC2713607

[B52] Swadlow H. Bezdudnaya T. Gusev A. (2005). Spike timing and synaptic dynamics at the awake thalamocortical synapse. Prog. Brain Res. 149, 91–105 10.1016/S0079-6123(05)49008-1 16226579

[B53] Swadlow H. Gusev A. (2001). The impact of ‘bursting’ thalamic impulses at a neocortical synapse. Nat. Neurosci. 4, 402–408 10.1038/86054 11276231

[B54] Thomson A. (1997). Activity-dependent properties of synaptic transmission at two classes of connections made by rat neocortical pyramidal axons *in vitro*. J. Physiol. 502, 131–147 9234202 10.1111/j.1469-7793.1997.131bl.x PMC1159577

[B55] Thomson A. Bannister A. (1999). Release-independent depression at pyramidal inputs onto specific cell targets: dual recordings in slices of rat cortex. J. Physiol. 519, 57–70 10.1111/j.1469-7793.1999.0057o.x 10432339 PMC2269491

[B56] Tsodyks M. Markram H. (1997). The neural code between neocortical pyramidal neurons depends on neurotransmitter release probability. Proc. Natl. Acad. Sci. U.S.A. Neurobiol. 94, 719–723 9012851 10.1073/pnas.94.2.719 PMC19580

[B57] Tsodyks M. Pawelzik K. Markram H. (1998). Neural networks with dynamic synapses. Neural Comp. 10, 821–835 9573407 10.1162/089976698300017502

[B58] Tsodyks M. Uziel A. Markram H. (2000). Synchrony generation in recurrent networks with frequency-dependent synapses. J. Neurosci. 20, RC50 10627627 10.1523/JNEUROSCI.20-01-j0003.2000 PMC6774142

[B59] Varela J. Sen K. Gibson J. Fost J. Abbott L. Nelson S. (1997). A quantitative description of short-term plasticity at excitatory synapses in layer 2/3 of rat primary visual cortex. J. Neurosci. 18, 7926–7940 9315911 10.1523/JNEUROSCI.17-20-07926.1997 PMC6793910

[B60] Varela J. Song S. Turrigiano G. Nelson S. (1999). Differential depression of excitatory and inhibitory synapses in visual cortex. J. Neurosci. 19, 4293–4304 10341233 10.1523/JNEUROSCI.19-11-04293.1999 PMC6782599

[B61] Wolfart J. Debay D. Le Masson G. Destexhe A. Bal T. (2005). Synaptic background activity controls spike transfer from thalamus to cortex. Nat. Neurosci. 8, 1760–1767 10.1038/nn1591 16261132

[B62] Zucker R. Regehr W. (2002). Short-term synaptic plasticity. Annu. Rev. Physiol. 64, 355–405 10.1146/annurev.physiol.64.092501.114547 11826273

